# Developing an Ensemble Predictive Safety Risk Assessment Model: Case of Malaysian Construction Projects

**DOI:** 10.3390/ijerph17228395

**Published:** 2020-11-13

**Authors:** Haleh Sadeghi, Saeed Reza Mohandes, M. Reza Hosseini, Saeed Banihashemi, Amir Mahdiyar, Arham Abdullah

**Affiliations:** 1 Department of Civil and Environmental Engineering, The Hong Kong University of Science and Technology, Clear Water Bay, Hong Kong, China; hsadeghiaa@connect.ust.hk (H.S.); srmohandes@connect.ust.hk (S.R.M.); 2 School of Architecture and Built Environment, Deakin University, Geelong 3217, VIC, Australia; reza.hosseini@deakin.edu.au; 3 Department of Building and Construction Management, University of Canberra, Bruce 2617, ACT, Australia; Saeed.Banihashemi@canberra.edu.au; 4 School of Housing, Building and Planning, Universiti Sains Malaysia, Penang 11800, Malaysia; 5 Universiti Malaysia Kelantan, Beg Bercunci No. 01, Bachok, Kelantan 16300, Malaysia; arham.a@umk.edu.my

**Keywords:** construction hazard, safety risk management, site management, data mining, neural network, fuzzy inference system, ANFIS, Malaysia

## Abstract

Occupational Health and Safety (OHS)-related injuries are vexing problems for construction projects in developing countries, mostly due to poor managerial-, governmental-, and technical safety-related issues. Though some studies have been conducted on OHS-associated issues in developing countries, research on this topic remains scarce. A review of the literature shows that presenting a predictive assessment framework through machine learning techniques can add much to the field. As for Malaysia, despite the ongoing growth of the construction sector, there has not been any study focused on OHS assessment of workers involved in construction activities. To fill these gaps, an Ensemble Predictive Safety Risk Assessment Model (EPSRAM) is developed in this paper as an effective tool to assess the OHS risks related to workers on construction sites. The developed EPSRAM is based on the integration of neural networks with fuzzy inference systems. To show the effectiveness of the EPSRAM developed, it is applied to several Malaysian construction case projects. This paper contributes to the field in several ways, through: (1) identifying major potential safety risks, (2) determining crucial factors that affect the safety assessment for construction workers, (3) predicting the magnitude of identified safety risks accurately, and (4) predicting the evaluation strategies applicable to the identified risks. It is demonstrated how EPSRAM can provide safety professionals and inspectors concerned with well-being of workers with valuable information, leading to improving the working environment of construction crew members.

## 1. Introduction

Occupational Health and Safety (OHS) in the construction industry, has not seen much improvement; thus, fatal and non-fatal occupational injuries occur frequently [1,2,3,4]. Due to the complicated nature of construction industry—continual changes, use of many different resources, temporary and transitionary employment, and harsh working environments—and involvement of various stakeholders, the number of occupational injuries in this industry is extremely high [2,5,6]. Given that workers play a key role in the construction industry, some human-related factors such as physical and mental health of the workers can play a pivotal role in occupational safety [7].

When it comes to OHS of workers, work-related injuries vary across industries and countries [8,9,10]. Countries with clear OHS regulations and requirements are quite different from those still in the process of adopting them. Taking Turkey as an example, the construction industry reported an average fatality rate of 22.35 in 100,000 workers, while figures for the manufacturing industry were around 6.2 between 2007 and 2016 [11]. Even more, the average fatality rate between 1988 and 2011 had jumped to 25.5 in the construction industry in Turkey [12]. Another developing country, whose construction industry constitutes a major proportion of fatal- and non-fatal accidents recorded among all industries, is Malaysia. Though the construction industry has recently evolved, thanks to the use of modern technology and equipment, yet the rate of accidents on construction sites has not noticeably decreased [13]. Hamid et al. [14] asserted that there was 125% increase of fatal injuries on construction sites in Malaysia from 2009 to 2015. Similarly, according to Muhamad Zaini et al. [13], fatalities on construction sites experienced an approximately 30% rise over a four-year period, starting from the year 2015. In contrast to developing counties, the status quo in developed countries is significantly better. Taking the US as an example, the fatal and non-fatal reported in 2017 were 3.1 per 100 people and 5.35 per 5190 people, respectively [11].

With the above in mind, constant improvement of current practices of the construction industry in developing countries should be high on the agenda for practitioners and policymakers alike. One remedial solution would be adopting appropriate and prudent assessment strategies [15]. Through having such propitious safety risk assessment, hazards and the ensuing accidents that pose threats to workers can successfully be identified, analyzed, and evaluated [16]. Considering this, various methods and techniques have been developed for risk analysis [17,18,19,20,21,22].

However, almost all available assessment models depend on considering two parameters, namely, probability and severity; these cannot lead to comprehensive assessments [23]. In addition, the use of raw numbers is a source of inconsistency, which cannot capture experts’ subjective views for conducting the assessment stage. Fuzzy Inference System (FIS), which maps the truth of any statement to a matter of a degree can overcome these shortcomings. Yet, one disadvantage of traditional FIS is that it often requires users to design the rules, which is impractical [24]. To solve this problem, Adaptive Neuro-Fuzzy Inference System (ANFIS) was developed by Jiang et al. [25], taking advantage of an artificial neural network (ANN) that is designed for learning characteristics and rules from large amount of data by adjusting the coefficients in the networks and reduce the error of output. Later on, ANFIS has been well-explored and widely adopted in safety management with proven long-lasting effects [26,27,28,29].

Despite the above developments, to date, there has not been any predictive safety risk assessment developed hitherto, through which identified risks can prudently be assessed using the integrated ANFIS method. Such scarcity leads to impairing the views of concerned safety professionals in dealing with the identified safety risks at later stages. To address these things, in this paper, an Ensemble Predictive Safety Risk Assessment Model (EPSRAM), based on the integration of neural networks with fuzzy inference systems, is developed to prudently assess the OHS risks related to workers on construction sites. Practicality of EPSRAM is showcased in applying it to several Malaysian construction projects. Following objectives are formulated in developing EPSRAM:

1. To fully identify crucial safety risks on construction sites, through interviewing several qualified experts; and
2. To analyze the magnitude of identified safety risks, and accordingly evaluate them using the proposed ANFIS method.

Findings reveal that where crucial safety risks are identified in construction sites, safety professionals will be provided with a prudent illustration of magnitudes, as well as, their suitable evaluations in an interactive and user-friendly way using the developed EPSRAM in the study. In other words, the major goal of the developed EPSRAM is to assist the concerned safety decision makers in predicting: (1) the magnitude of safety risks identified, and (2) the suitable evaluation strategy for dealing with the safety risks analyzed.

The remainder of this paper is designed as follows. Section 2 provides a brief review of previous studies on the topic, and Section 3 delves scrupulously into EPRAM, as developed. Section 4 is allocated to share the findings accompanied by discussions. Section 5 is for acknowledging limitations and offering recommendations for future studies, whereas concluding remarks are presented in Section 6.

## 2. Literature Review

Assessing safety risk in construction sites has received much attention from researchers over the past decade. Hallowell [30] proposes a new model that evaluates safety and health risk of construction processes and can be used as a systematic method for strategic selection of safety program elements, with a database of industry experts’ experience in construction risk prevention [30]. The author quantifies construction risk with probability and severity, to evaluate the risk of construction activities or safety program elements. With rapid advancement of computing power and related methods in recent years, different models and programs on assessing construction risk are introduced with improved accuracy. For example, Aminbakhsh et al. [18] proposed a security risk assessment framework based on the safety cost theory (COS) model and analytic hierarchy process (AHP), to address construction budget overrun, without compromising worker’s safety [18]. Since construction activities are interrelated in several aspects and executed simultaneously, Esmaeili and Hallowell [31] developed a decision support system to assess the safety level of workers with Delphi method. In a study conducted by Sousa et al. [32], a model was developed for the enhancement of workers’ safety for resource management. A statistic model was developed by Isaac and Edrei [33] to monitor the exposure of workers for various activities. In order to develop this model, they used a series of data related to the duration of activities.

Fatalities have been reported as a safety serious issue related to construction activities; as a result, several research studies have focused on this issue. For instance, Malekitabar et al. [34] investigated the risk of accidents—considering probability and severity—by identifying five-level safety drivers. Tixier et al. [35] developed a model to analyze the safety risks by Kernel density estimators and Copulas, while Raviv et al. [36] especially analyzed accidents that occur in using tower crane. When it comes to the adoption of sustainable approaches in the construction industry, safety requirements need to be met to ensure every possible new safety risk is carefully considered. Therefore, several research studies have been conducted to assess safety risks using sustainable techniques (e.g., Dewlaney et al. [37], Karakhan and Gambatese [38], Hwang et al. [39], and Zhang and Mohandes [40]).

In another stream of research, probabilistic approaches have been widely used for the purpose of safety risk assessment in the construction industry. For example, Mahdiyar et al. [41] used Monte Carlo simulation for the safety assessment of slopes under seismic conditions. However, this approach requires a large dataset, which is not typically available on worker’s safety. As a result, the use of experts’ knowledge and fuzzy set theory has been recommended rather than statistical- and probabilistic- based models [42,43], to deal with the insufficiency of and uncertainties of data. There are many studies on developing risk assessment models or decision support systems, considering different conditions and parameters and using variety of methods. One example is the integration of Pythagorean fuzzy analytic hierarchy process with different Multi-Criteria Decision-Making (MCDM) methods. (e.g., [15,44,45,46,47,48,49,50,51,52,53]).

Despite extensive research and improvements in recent years, uncertainty is usually the main cause of model failures. Besides, collecting data from construction industry experts is still problematic, given that any data collected from experts are exposed to subjective judgments, with high variance. Therefore, a model that can accurately predict construction risks is much needed. As mentioned above, a study in which a predictive model for safety risk assessment is developed is nonexistent. More importantly, the topic of safety risk assessment in Malaysia has not received adequate attention from researchers, despite the cruciality of this matter in developing countries like Malaysia. Thus, developing a comprehensive predictive assessment model is very relevant, particularly to the Malaysian context, where practitioners still rely on traditional assessment methods. As such, this study intends to propose a model for prediction of occupational risks that affect construction workers based on experts’ perceptions using ANFIS method. Through this—proposed EPSRAM—construction managers can assess the risk factors related to construction projects with higher efficiency compared to conventional risk assessment methods, which involve a large number of subjective judgments from construction project experts.

## 3. Research Methods

**Figure 1:**
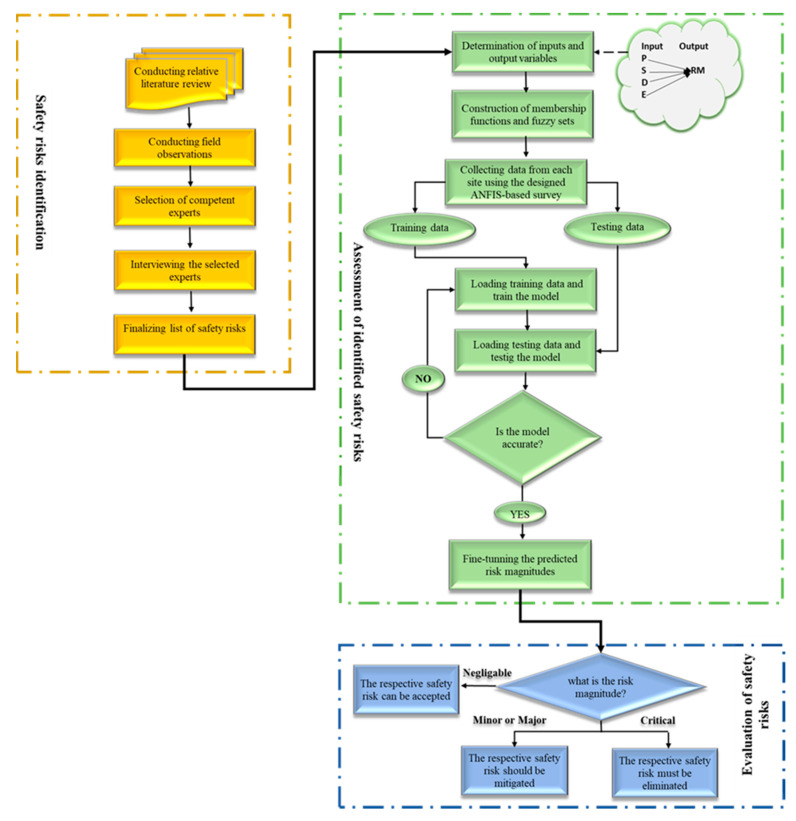
The developed EPRAM.

Figure 1 illustrates the developed EPSRAM. This includes three main stages, namely identification of safety risks, ANFIS exploitation, and evaluation of analyzed risks, as discussed next.

### 3.1. Identification of Safety Risks

**Figure 2:**
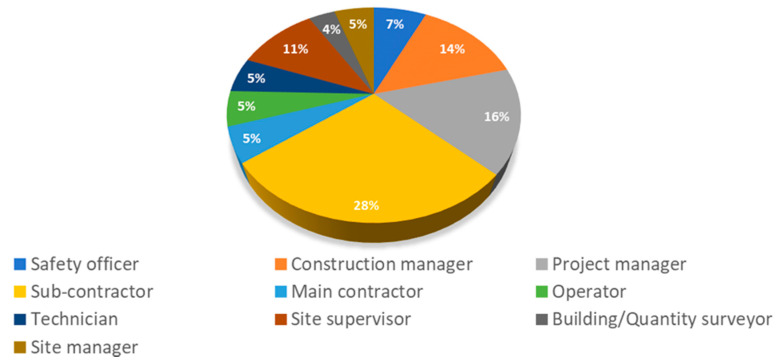
The breakdown of interviewed experts.

As the first step, a comprehensive and detailed list of safety risks posing threats to the construction workers on Malaysian construction sites was needed. To this end, a meticulous review of the current literature was considered—papers, reports, articles, and so forth. Afterward, structured interviews with experts were conducted. In doing so, safety officers were interviewed to identify the safety risks occurred on construction sites, either through their firsthand experience or any records they had come across in reports and documents, as mentioned in Zhang and Mohandes [40]. Afterwards, the prepared list of risks—obtained from safety officers—was presented to the experts who had at least five years of related experience and a related bachelor’s degree on an area related to construction engineering and management (see Figure 2). These experts were interviewed, where they were asked to add any more risks that had not been considered. This resulted in a comprehensive list of risks. Noteworthy to mention is that many experts from different backgrounds were interviewed. That is, fifty-seven experts who worked in seventeen different construction sites in Kuala Lumpur were involved, in order to increase the reliability and coverage of results.

### 3.2. Assessment of Identified Safety Risks

Once the detailed list of safety risks had been compiled at the previous stage, then field observation of construction sites for the sake of their assessment using the ANFIS model was considered. Of the selected ongoing construction projects, ten sites (whose constructors or developers were willing to contribute to the study) were taken into consideration. Table 1 shows the profile of experts working on these construction sites. In order to fully consider all the essential risk parameters based on which safety of construction workers can be assessed, a meticulous investigation into previous studies was carried out [47,48,54,55,56]. Ultimately, four crucial parameters, which can collectively assess the level of a safety risk posing danger to workers, were identified, being probability, severity, exposure, and detectability. Probability is concerned with the possibility of occurrence of a risk, while the impact of any risk occurred to a worker is called severity. The duration of the worker’s exposure to the respective hazardous situation is regarded as exposure. Detectability unravels the extent to which workers can detect the occurrence of safety risk.

With the above in mind, Equation (1) illustrates the final Risk Magnitude (RM):

(1) RM=p×s×e×d

where p, s, e, and d represent probability, severity, exposure, and detectability, accordingly. It is essential to mention that the input data (P, S, E, and D) and output data (RM) were normalized to simplify the procedure using Equation (2), which has been used by many researchers [57,58].

(2) $$X_{n}=\frac{X_{i}-X_{min}}{X_{max}-X_{min}}$$

During field observations, chosen experts were asked to assign linguistic variables to each safety risk identified with regard to each risk parameter separately (see Table 2, Table 3, Table 4 and Table 5). As can be seen from these Tables, the appropriate normalized values—with respect to each variable—for further analysis using ANFIS technique are provided. Notably, one expert from each site was selected, who had more related experience. As such, 290 (i.e., based on the multiplication of twenty-nine risks by ten sites) data sets were collected from field observations, of which 203 and 87 were used for training and testing, respectively. The amount of data collected were deemed prudent enough for conducting ANFIS-based analysis, as mentioned in Fragiadakis et al. [28]. Note that Appendix A illustrates the sample of questionnaire presented to each expert on selected construction sites. A brief exposition on the concept of Fuzzy Inference System (FIS) together with the structure of ANFIS method are provided next.

#### 3.2.1. Fuzzy Inference System

Fuzzy Inference System (FIS) is a decision-making methodology, which takes input values and creates output fuzzy values based on some logic rules [59,60]. Sometimes decisions need to be made based on real-world observations (which can be crisp or fuzzy values) and some fuzzy logic rules (i.e., IF-THEN rules) [26]. Technically, there are two types of Fuzzy Inference Systems, such as Mamdani Fuzzy Inference System and Takagi-Sugeno Fuzzy Model. In this paper, a variant of Takagi-Sugeno Fuzzy Model called ANFIS is adopted to analyze safety risks [61], as discussed below.

#### 3.2.2. ANFIS

One disadvantage of traditional Fuzzy Inference System (FIS) is that it often requires users to design the rules, which is sometimes impractical because in some decision-making problems, the relationship between inputs and outputs are not clear and there are no intuitive methods to design the rules [57]. Meanwhile, artificial intelligence-based methods are attracting more and more attention and the artificial neural network (ANN) is a model that can learn characteristics and rules out of large amount of data. ANN works by adjusting the coefficients in the networks with the aim of reducing the error of output [57].

Jiang et al. [25] combined FIS with neural networks in 1993 in order to overcome the shortcomings of FIS and introduced Adaptive Neuro-Fuzzy Inference System (ANFIS). ANFIS is essentially a neural network with five layers. If there are two inputs parameters, z and y, and accordingly one output f, then the constructed rules can be shown as Equations (3) and (4).

(3) $$\mathrm{If}\;z\;\mathrm{is}\;M_{1}\;\mathrm{and}\;y\;\mathrm{is}\;N_{1},\;\mathrm{then}\;f_{1}=m_{1}z+n_{1}y+p_{1}$$

(4) $$\mathrm{If}\;z\;\mathrm{is}\;M_{2}\;\mathrm{and}\;y\;\mathrm{is}\;N_{2},\;\mathrm{then}\;f_{2}=m_{2}z+n_{2}y+p_{2}$$

where, $M_{1},M_{2},N_{1}\;\mathrm{and}\;N_{2}$ are the fuzzy sets and $f_{1}\;\mathrm{and}\;f_{2}$ are the resultant output in this example. Furthermore, the value of design parameters $z_{i},\;y_{i}\;\mathrm{and}\;p_{i}$ can be identified when the training process is executed. The main reason why the ANFIS technique is used in the current study relies upon the fact that the magnitude of safety risks as well as their related evaluation strategies can be predicted, in particular when the observed data lacks or are incomplete. In a nutshell, the four essential risk parameters are considered as inputs to the ANFIS technique, while the magnitude of safety risks are the outputs. Through such developed model, the practitioners can predict the criticality level of risks, based on the characteristics and contextualization of the relative sites under the investigation. This is, to the best of authors’ knowledge, the first attempt to incorporate machine learning into the area of construction safety risk assessment. The detailed steps of ANFIS exploitation are explained in the following lines.

The use of ANFIS entails 6 steps:

Step 1. Data collection: The purpose of this step is to collect a dataset including inputs and corresponding outputs for training and testing. Four variables or risk parameters (namely probability, severity, exposure, and detectability) and one variable (i.e., risk magnitude) are selected as input and output variables, respectively.

**Figure 3:**
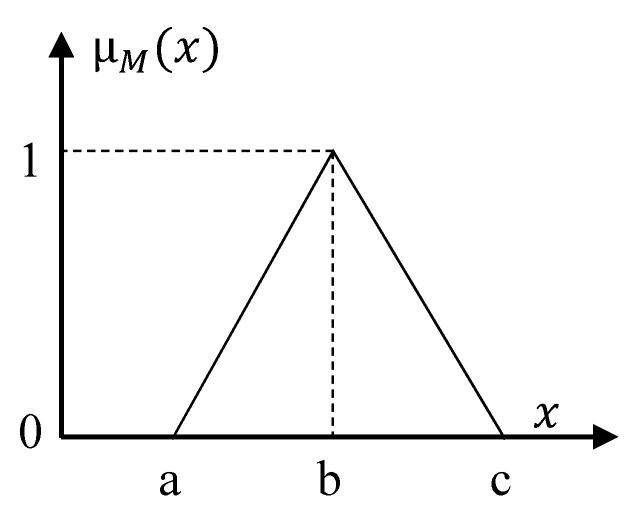
Triangular fuzzy set membership function.

Step 2. Construct Fuzzy sets: ANFIS is a fuzzy inference system, in which membership functions and fuzzy sets should be defined in the preparation process of ANFIS, as adopted in this paper to identify fuzzy sets for output variable (Risk Magnitude). Notably, as mentioned by Ponce et al. [61] and Chang et al. [62], the most proper type of fuzzy interface system for adaptive approaches is Takagi-Sugeno Fuzzy Model. Because the triangular membership function is one of the simplest among the other types and can be easily applied to the parameters, Triangular Membership Function is used in this paper, as in Equation (5).

(5) $$Triangular\left(x;a,\;b,\;c\right)=\{\begin{array}{c} 0,\;x<a\\ \frac{x-a}{b-a},\;a\leq x\leq b\\ \frac{c-x}{c-b},\;b\leq x\leq c\\ 0,\;d\leq x \end{array}$$

where a, b, c represent the lower, median, and upper bounds of fuzzy sets, which are user parameters. Each linguistic variable of each input corresponds to a fuzzy set and has a triangular membership function with different parameters (i.e., a, b, c). Figure 3 shows the triangular membership function graph.

If the collected data are in the form of linguistic data, each linguistic input will need to be normalized by converting them into numerical values using Equation (2).

Step 3. Divide the Dataset into Training and Test Datasets: In order to train the ANFIS model, as well as evaluate the quality of the trained model, two datasets are needed in the form of training set and test set. These are obtained by dividing the overall dataset into two parts.

Step 4. Train the ANFIS model: This step is comprised of five levels, which are needed for training the Sugeno-type FIS, as explained next. As discussed before, ANFIS outperforms traditional Fuzzy Inference Systems—does not require given rules. ANFIS can learn the rules from the large amount of training data. Since the rules are encoded in layer 4 of ANFIS, coefficients in this layer need to be trained in order to represent the rules in ANFIS. To train the ANFIS with the training dataset generated in a previous step, coefficients in layer 4 are initialized with random numbers and inputs in the dataset given to ANFIS to calculate an output. The calculated output is then compared with the ground truth output in the dataset and the coefficients are adjusted based on the errors that ANFIS calculates for the output. There have been many approaches to adjusting the coefficients based on the output errors. Of these, the most frequently used approaches are least square method and back propagation algorithm. Notably, in this research, forward propagation and least squares estimation are used as learning algorithm for parameters associated with input and the output membership functions, respectively.

##### Fuzzification Layer (Layer 1)

The first layer of ANFIS is used to fuzzify the input values using membership functions. The output of this layer is used as the input of layer 2. The output of fuzzification layer is calculated by Equation (6):

(6) $$L_{1}^{i}=\mu_{M_{i}}\left(z\right)$$

where $\mu_{M_{i}}\left(z\right)$ denotes the membership function for input z and linguistic variable $M_{i}.$

##### Product Layer (Layer 2)

**Figure 4:**
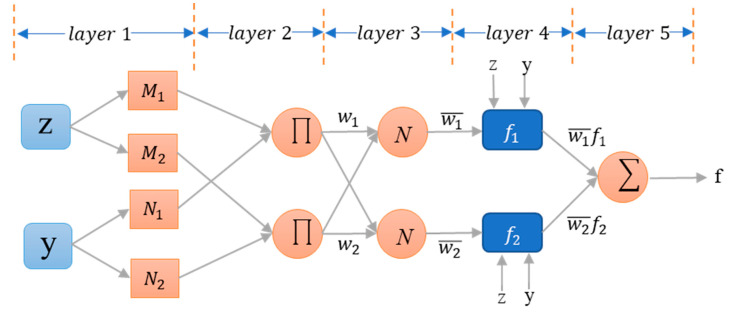
The ANFIS architecture with two inputs (z, y), two rules, and one output (f).

This layer calculates the firing strength of the rule (the weight). As shown in Figure 4, the membership function weights ($w_{i}$) is calculated based on Equation (7):

(7) $$L_{2}^{i}=w_{i}=\mu_{M_{i}}\left(z\right)\;\times \mu_{N_{i}}\left(y\right)$$

where $w_{i}$ stands for the rule weights, which is used as the input of layer 3. Notably, in ANFIS, the weights of rules are equivalent to weights of biases in the traditional ANN [26].

##### Normalized Layer (Layer 3)

In this layer, ANFIS normalizes the weight values obtained from layer 2 using Equation (8):

(8) $$L_{3}^{i}={\bar{w}}_{i}=\frac{w_{i}}{\sum_{i=1}^{4}w_{i}}$$

The aim of normalization is to replace each weight value with its ratio in the sum of all weights. By normalization, one can constrain the weight value ranges into [0, 1].

##### De-fuzzification (Layer 4)

In the defuzzification layer, the weighted output is computed through the multiplication of calculated normalized weight (${\bar{w}}_{i}$) by the result of the linear regression model associated with the current node as Equation (9):

(9) $$L_{4}^{i}={\bar{w}}_{i}f_{i}={\bar{w}}_{i}\left(m_{i}z+n_{i}y+p_{i}\right)$$

where ${\bar{w}}_{i}f_{i}$ and $f_{i}$ represent the weighted output and output of i^th^ rule, respectively. Furthermore,$m_{i}$, $n_{i}$ and $p_{i}$ are associated parameters. These coefficients encode the rules; they are obtained via the training process of ANFIS.

##### Output Layer (Layer 5)

The overall output of ANFIS is generated in this layer using Equation (10):

(10) $$output=L_{5}^{i}=\overset{4}{\underset{i=1}{\sum }}{\bar{w}}_{i}f_{i}=\frac{\sum_{i=1}^{4}w_{i}f_{i}}{\sum_{i=1}^{4}w_{i}}$$

where ${\bar{w}}_{i}f_{i}$ is the outputs from the previous layer (i.e., layer 4).

Step 5. Evaluate the model: In this step, the test dataset generated in Step 3 is used to evaluate the output of ANFIS by comparing the ground truth output in the dataset and the output calculated by ANFIS. Since the test dataset is disconnected from the training dataset, the assessment with test dataset can evaluate the generalization capability of the fuzzy inference system.

Step 6. Verify the accuracy of the Model: To further verify the accuracy of prediction of ANFIS, some performance indices like RMSE (root-mean-square error) and MAPE (mean absolute percentage error) are used. Using these performance indices, it can be assessed if the prediction made by ANFIS is consistent with the real output or otherwise [63]. These three performance indices are presented as in Equations (11)–(13):

(11) $$RMSE=\sqrt{\frac{1}{n}\overset{n}{\underset{i=1}{\sum }}{\left(a_{i}-c_{i}\right)}^{2}}$$

(12) $$MAPE=\frac{1}{n}\overset{n}{\underset{i=1}{\sum }}\left|\frac{a_{i}-c_{i}}{a_{i}}\right|$$

(13) $$R^{2}=\frac{{\left[\sum_{i=1}^{n}\left(a_{i}-{\bar{a}}_{i}\right)\times \left(c_{i}-{\bar{c}}_{i}\right)\right]}^{2}}{\sum_{i=1}^{n}\left(a_{i}-{\bar{a}}_{i}\right)\times \left(c_{i}-{\bar{c}}_{i}\right)}$$

In these equations, $a_{i}$ and $c_{i}$ denotes the measured and predicted output, accordingly. The ${\bar{a}}_{i}$ and ${\bar{c}}_{i}$ stand for the average of predicted and measured data. Additionally, *n* represents the number of samples, by which the prediction accuracy is assessed.

### 3.3. Evaluation of Safety Risks

In any comprehensive safety risk assessment model, the final stage is all about the selection of appropriate evaluation strategies to address the analyzed safety risks [53]. To this end, the proposed EPRAM in this paper takes into account three levels of evaluation strategies using a linear interpolation technique, as suggested by Mohandes et al. [45]. In doing so, the maximum and minimum values (which equal to respectively 1 and 6561 in a study conducted by Karasan et al. [48]) were normalized within the range of [0, 1]. Table 6 shows the mentioned range of values along with related magnitudes, considering the following rules:

- If the predicted magnitude (output of the model) is negligible, then it should be accepted, that is, the respective safety risk can be accepted, provided that its magnitude is not increased to a higher level over the reassessment stage
- If the predicted magnitude is either minor or major, then it should be mitigated: the safety risk magnitude should be reduced to the threshold value, as agreed upon by project managers
- If the predicted magnitude is critical, then it should be eliminated, that is, all related hazardous activities should be stopped until it is reduced to the lower level (i.e., medium, major, or negligible).

Notably, the major reason for the adoption of linear interpolation technique lies in the fact that the parties concerned with safety and health of construction workers can come up with the suitable evaluations for controlling the safety risks analyzed in an easy and user-friendly way.

## 4. Results and Discussion

**Figure 5:**
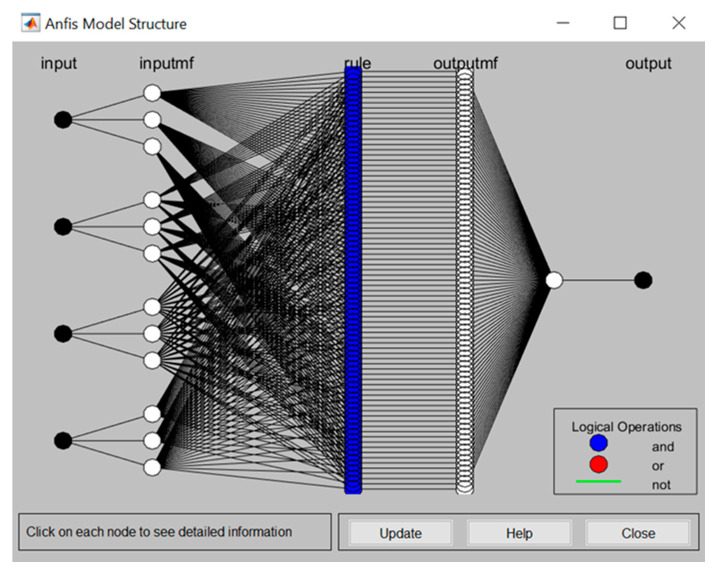
Structure of used ANFIS model.

**Figure 6:**
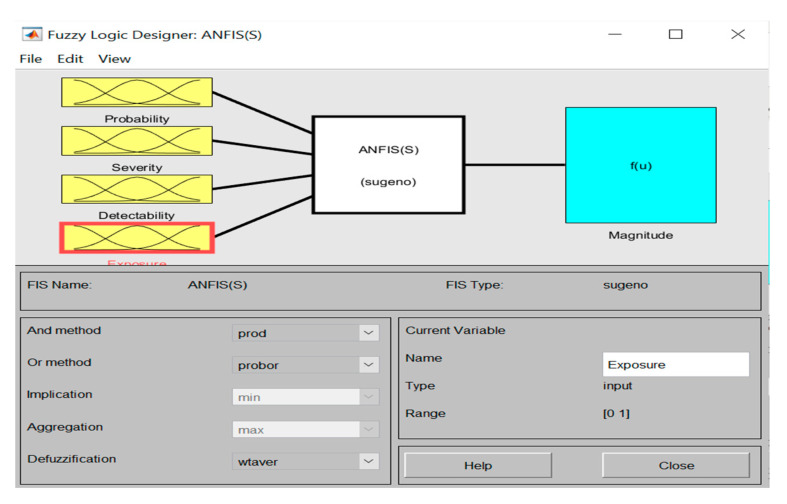
Inputs and output in ANFIS.

As discussed, this study delves into the assessment of safety risks that pose threats to construction workers in Kuala Lumpur. For the sake of brevity, only the main results are reported hereinafter. Firstly, through interviewing the qualified experts selected for the study, a comprehensive pool of crucial safety risks was developed, as tabulated in Table 7. Subsequently, based on the data collected from the chosen construction sites (which are related to the assessment of safety risk regarding the four determined parameters), several fuzzy-based rules were created using Sugeno-type FIS method. Table 8 summarizes the data from one of the selected construction sites. To check and approve the consistency of results, Cronbach alpha was used as recommended by Zhang and Mohandes [40], as a result of which responses were found to be of good consistency—the calculated alpha equals 0.7973. With the consistency of collected data established, four rules were then built up based on the variables assigned to the defined safety risk. To take SR1 as an example, the following rules were created in MATLAB (Version 2017b): If P is M, S is VH, E is M, and E is M, then $f_{1}=0.171$. Similarly, the pertinent rules for other safety risks on each construction site were created. Figure 5 and Figure 6 illustrate respectively the whole structure of the developed ANFIS model and the fuzzy logic designer page for the inputs to predict the desired outputs. To run the simulations related to the proposed ANFIS method, ANFIS toolbox in MATLAM was used, in view of its ability in providing interactive as well as inclusive results [26,28].

**Figure 7:**
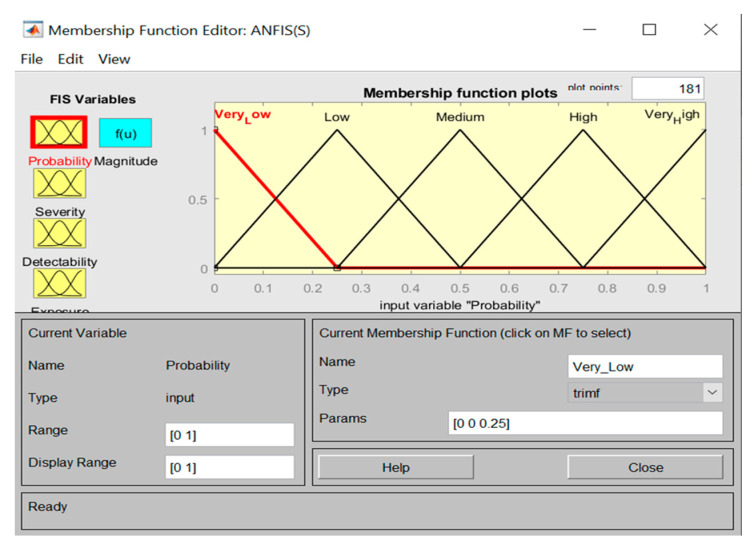
Triangular membership functions with membership values between 0–1.

Furthermore, Figure 7 demonstrates the fuzzy ratings and the membership functions for inputs (i.e., triangular membership functions).

**Figure 8:**
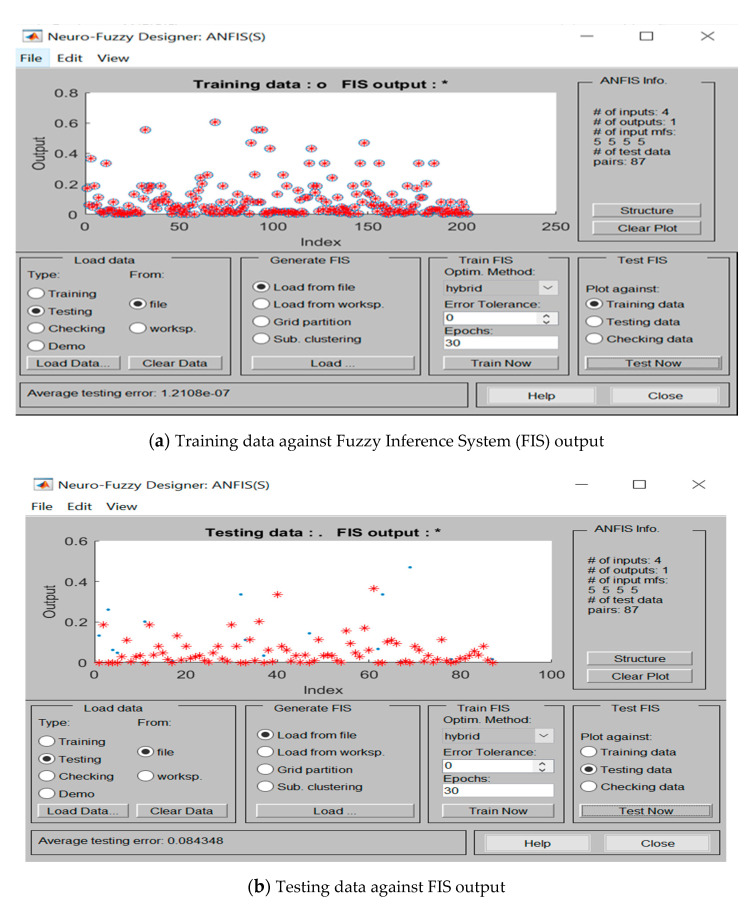
Performance of the Adaptive Neuro-Fuzzy Inference System (ANFIS) model on (**a**) training, and (**b**) test data.

As inferred from Figure 8, the trained and tested output of ANFIS, which illustrates the predicted Risk Magnitude (FIS output) for both training and testing data, are in good harmony with the actual data. Notably, after training the pertinent data set in ANFIS, it was observed that the training process ceased at epoch 12. Table 9 shows the ANFIS structure together with related training parameters.

**Figure 9:**
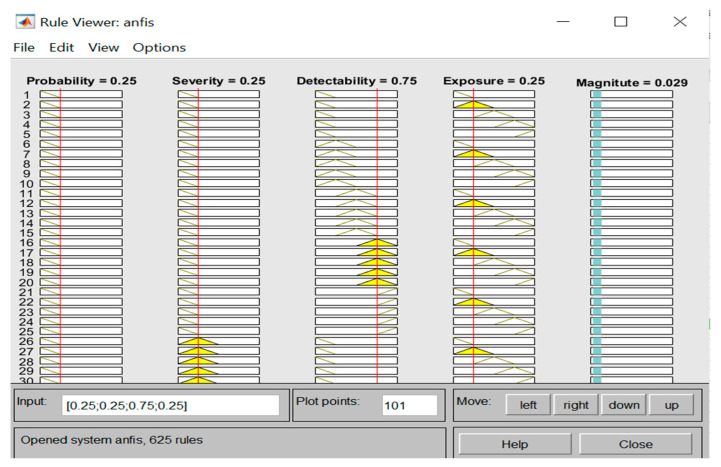
Rule viewer created for the training datasets.

**Figure 10:**
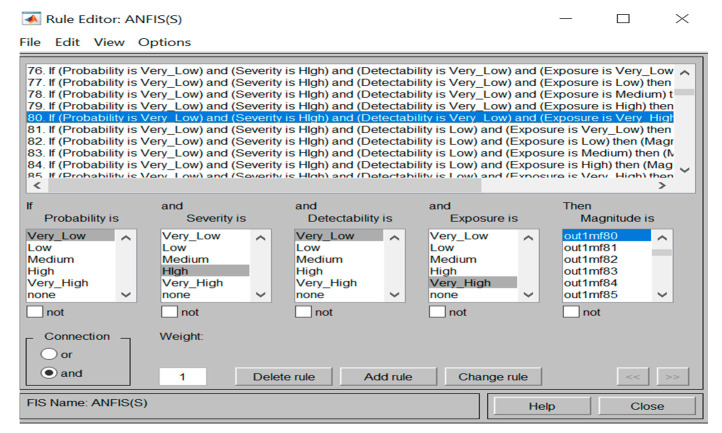
The constructed rules by ANFIS Sugeno.

Furthermore, once the 203-training data has been fed to the developed ANFIS Sugeno model, the rules were then automatically created as can be seen from the rule viewer (see Figure 9 and Figure 10). Consequently, based on the training data set fed to the ANFIS model, the magnitude of the safety risks for the testing step (87 datasets that were garnered from three construction projects) was predicted, as shown in Table 10.

**Figure 11:**
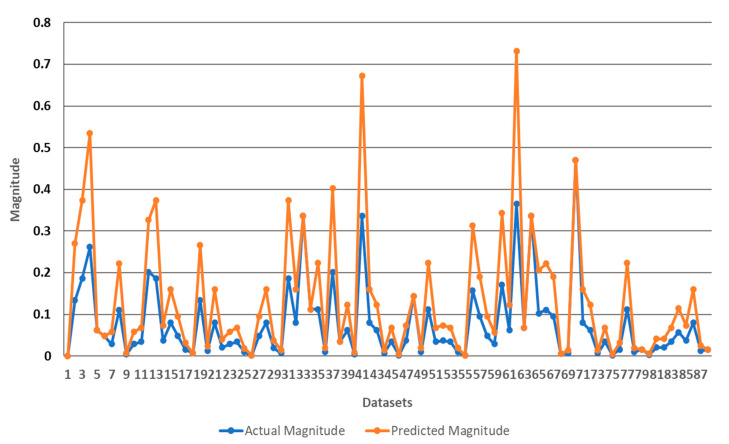
The comparison between actual versus predicted risk magnitudes of testing data sets.

**Figure 12:**
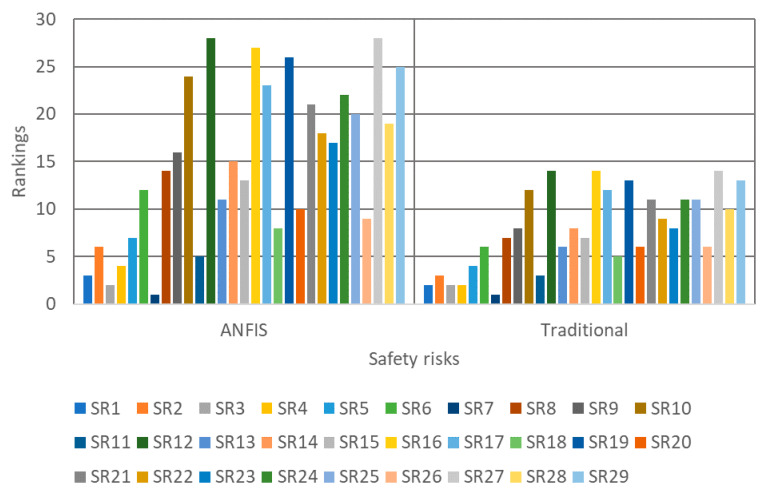
Results of comparative analysis.

As illustrated in Table 10, a comparison of 29 datasets (i.e., collected from one construction site) of actual data (i.e., testing data) and predicted value by ANFIS Sugeno is available. Moreover, the efficiency of the optimization model can be seen in Figure 11, which illustrates the comparison of predicted and recorded risk values of 87 testing data sets. Furthermore, to show the accuracy of the used ANFIS model in the study for assessing the identified safety risks, the results obtained were compared against those of Linear Regression Method (LRM). As in Table 11, the outcomes of ANFIS model are more accurate according to the different performance measures considered. Additionally, a comparative study between the rankings obtained from the proposed EPRAM and the traditional assessment (in which two parameters are used, namely probability and severity) was considered. In doing so, the assessments, which were related to the exposure and detectability parameters, were neglected, and accordingly, the remaining inputs were fed to the proposed ANFIS model. Figure 12 shows the rankings obtained from both models. As can be observed, many safety risks were placed in the same spots using the parameters considered in traditional assessment method. Hence, the concerned safety professionals cannot take the further prudent mitigative strategies, since the analyzed safety risks cannot be distinguished from each other. To make it more explicit, using the traditional assessment approach (that are rampant in the construction industry), some risks are ranked in the same spots. For instance, as can be observed from Figure 12, SR1, SR3, and SR4 all are ranked in the same place, for which the same mitigative strategy should be come up with; hence, achieving prompt responses for dealing with the riskier hazards cannot come to fruition. By contrast, the project managers and decision makers can appropriately prioritize the mitigative measures according to the ranking of safety risks. Looking at the aforesaid example, SR3, SR1, and SR4 should sequentially be treated (based on their final rankings). In a nutshell, the obtained diversified rankings through the proposed EPSRAM leads to making the concerned parties understood of the criticality levels of safety risks analyzed (the higher the final ranking of risk, the earlier the relevant responsive measure).

It is notable that based on the predicted RM of each safety risk, the proposed evaluation strategies (mentioned in Table 6) were taken into account, and accordingly the suitable actions with regard to the risks analyzed along with their final rankings are tabulated in Table 11.

As inferred from the results, a majority of safety risks identified is of minor or major magnitude, for which the appropriate mitigative measures should be considered by safety decision makers and regulators. Additionally, it was witnessed that the operations associated with twelve risks need to be stopped (since their magnitude falls into the critical level), until the pertinent magnitudes are reduced. Another noticeable observation was related to the ranking of those safety risks identified on the selected construction sites: top five risks are SR7 (being struck by falling objects), SR3 (being trapped), SR1 (being shocked), SR4 (fall from height), and SR11 (spinal disc injury). Even though the order of criticality levels of top-ranked safety risk identified in the study are slightly different from the observations mentioned in previous studies (which is due to consideration of a higher number of risk parameters), the overall picture is to a large extent the same. For instance, Hamid et al. [14] opined that fall from height, electrocution, and struck by falling objects are the main culprit of fatal-related injuries on Malaysian construction sites. Similarly, the perilousness of being trapped by the machineries was emphasized by Chong and Low [2]. Likewise, Torghabeh et al. [64] stated that the safety professionals in Malaysia are concerned with the ergonomic-pertinent injuries, as the majority of respective construction workers suffer from these non-fatal injuries through their careers. The main reason behind this lies in the fact that ergonomic-related injuries are not usually reflected in related databases or archives, since they mostly do not account for any direct fatalities; however, their direct or indirect impacts on workers or their family members can be disastrous.

Having said that, considering the full picture of essential risk parameters reveals the fact that there are many other critical safety risks that have not received attention in previous studies. Damages to the tendons (SR13), stenosing tenosynovitis (SR14), neck stiffness (SR15) as few examples, these safety risks were observed to have been eliminated, since their magnitudes are observed to be critical. That said, these safety risks in the related literature are not referred to. The major reason for their omission lies in the fact that identified risks have been typically explored from two aspects: whether their occurrences were possible or not (i.e., probability), and whether the resulting impacts were noticeable or not (i.e., severity). In fact, the results of previous safety assessment of risks in Malaysian construction sites have overlooked two crucial aspects of a hazard, namely the duration of exposure, as well as the detection of occurrence of a risk by workers. As a result of these disregards, many impactful safety risks have been overlooked. Therefore, moving beyond the boundaries of common parameters considered in traditional assessment approaches is a dire need of the construction industry. Working environments for construction workers can be safer by considering a wide range of hazardous situations and extending common parameters.

Moreover, during the interviews at the initial stage of the research, it was observed that lack of proper supervision by main contractors is a major cause for many highlighted safety risks. Similarly, the existence of loopholes in regulations, and most importantly, the flawed safety management systems within construction firms are behind many safety problems on sites. Furthermore, it was unanimously asserted that through (1) providing appropriate safety training for workers, as well as (2) reducing their exposures to hazardous situations by utilizing modernized equipment, the occurrence of many critical ergonomic-related risks can be contained.

## 5. Limitation and Recommendations

Although the proposed model in this paper led to the obtainment of precise and inclusive results for the assessment of safety risks, there are some limitations to be acknowledged:

1. Although the exploitation of experts’ points of view as the main source of data collection in this study has led to building up a predictive safety assessment model (that can predict the magnitude of risks as well as their evaluations with great accuracy), it is recommended to conduct a research using recorded actual data on sites; so that the results can be compared against those of current research findings.
2. To increase the generalizability of results, the proposed model needs to be applied to a wide range of case studies. Application of the developed EPRAM to different sites provides a fertile ground for future research.
3. The developed integrated model in the study lacks a proposal for further mitigative actions of the risks. As such, there is a need to develop treatment strategies for the safety risks evaluated in the proposed EPRAM. This will provide safety professionals with a better picture in dealing with safety risks.
4. Last but not the least, although the proposed model can capture the uncertainty in experts’ perceptions, it cannot handle reliability levels in the assessments provided by respective safety experts. Thus, integration of Z-numbers-based technique with neural networks remains a promising area for researchers interested in the exploitation of machine learning techniques and their applications to construction safety domain, for obtaining more inclusive results.

## 6. Conclusions

Despite the extensive corpus of literature on OHS assessment of construction workers, there has been a dearth of study that incorporates the idea of machine learning into the existing body of knowledge. In addition to this gap, no systematic safety risk assessment investigation to date has been fostered on Malaysia construction projects, where the respective sector is evolving at a brisk pace. To fill these gaps, an innovative EPRAM is developed in this study. Through applying the developed EPRAM to ten construction projects in Kuala Lumpur, this paper contributes to the body of knowledge in several ways, as explained below.

1. *Overcoming the shortcoming existing in the traditional-based assessment methods*: To conduct traditional-based assessment methods, large amounts of data on OHS-related issues must be collected. The data are subject to errors in various ways including incomplete archived data, as well as legal issues of sharing the data. Using the fuzzy rule-based system reported in this paper, these shortcomings are resolved.
2. *Developing a conclusive OHS assessment with four essential risk parameters*, as opposed to traditional-based assessment approaches, which are based on only two parameters—probability and severity. Applying the proposed EPRAM to a number of construction projects in Malaysia, it was observed that apart from well-known critical safety risks (namely fall from heights, struck by falling objects, electrocution, trapped), there are some types of risks that have not yet been even pointed out. For instance, overexertion-related injuries were witnessed to be of high criticality (such as spinal injury, damages to tendons, neck stiffness, and so forth), demanding more attention from safety professionals. One way to handle these types of risks is through providing appropriate safety training for workers, as well as reducing their exposures to the related hazardous situations by utilizing modernized equipment. There are overlooked in traditional risk assessment methods.
3. *Overcoming the problems associated raw numbers* that being used in traditional-based assessment methods by fuzzifying the relative data is another novel feature of this paper. With this, the final output—risk magnitude of risks—are of high accuracy. The results produced were observed to outperform traditional-based safety assessment methods, as well as linear regression methods.
4. *Proposing appropriate evaluation strategies* based on predicted magnitude values of risks. That is, through the developed EPRAM model in the study, safety professionals are provided with suitable measures to deal with safety risks based on the newly-defined classifications in the study.

It is hoped that the developed predictive model in the study can guide authorities and professionals towards perceiving better illustration of safety risks identified, by providing them with the obtainment of risk magnitudes, as well as how to evaluate them later on, in an easy and interactive way. This, in turn, will improve the OHS of the workers embroiled in the relative construction activities. On the other side of the coin, when it comes to the practicality aspect, it has to be said that the application of the developed model is intended to be as user-friendly as possible for the concerned parties, including safety inspectors, project managers, construction managers, site supervisors, safety officers, and so forth. That is to say, it can be applied to new construction projects by feeding more data into the proposed prototype; however, more research should be conducted towards its commercialization for wider adaptability. Having said that, developing an innovative mitigative model, which includes the proposal of treatment measures for the risks analyzed and evaluated using the proposed model in this research, seems an undeniable fact for the future studies. Additionally, the integration of Z-numbers with neural networks could be regarded as a potential future stream, and accordingly, the results can be compared with one another.

## Figures and Tables

**Table 1:** Experts’ profile.

NO. of Expert	Position	Experience (Years)	Degree	Selected Construction Sites’ Characteristics
1	Sub-contractor	Between 15 and 20	Undergraduate in civil engineering	Medium-sized
2	Construction manager	Between 15 and 20	Master’s in construction management	Large-sized
3	Site supervisor	Between 10 and 15	Undergraduate in civil engineering	Medium-sized
4	Sub-contractor	Between 15 and 20	Undergraduate in civil engineering	Medium-sized
5	Sub-contractor	Between 15 and 20	Undergraduate in civil engineering	Medium-sized
6	Sub-contractor	Between 10 and 15	Master’s in construction management	Medium-sized
7	Construction manager	Between 15 and 20	Master’s in project management	Medium-sized
8	Safety officer	More than 20	Undergraduate in civil engineering	Medium-sized
9	Main contractor	Between 10 and 15	Undergraduate in quantity surveying	Large-sized
10	Sub-contractor	Between 10 and 15	Undergraduate in quantity surveying	Medium-sized

**Table 2:** Linguistic variables for probability.

Scales (Definitions)	Normalized Value	TFNs
Very low (the chance of occurrence of relative risk is almost impossible)	0	(0, 0, 0.25)
Low (the chance of occurrence of relative risk is negligible)	0.25	(0, 0.25, 0.5)
Medium (the chance of occurrence of relative risk is expected)	0.5	(0.25, 0.5, 0.75)
High (the chance of occurrence of relative risk is quite possible)	0.75	(0.5, 0.75, 1)
Very High (the chance of occurrence of relative risk is almost certain)	1	(0.75, 1, 1)

**Table 3:** Linguistic variables for Severity.

Scales (Definitions)	Normalized Value	TFNs
Very low (if the relative risk occurs, the resultant injury level of the workers is first aid)	0	(0, 0, 0.25)
Low (if the relative risk occurs, the resultant injury level of the workers is minor)	0.25	(0, 0.25, 0.5)
Medium (if the relative risk occurs, the resultant injury level of the workers is minor disabilities)	0.5	(0.25, 0.5, 0.75)
High (if the relative risk occurs, the resultant injury level of the workers is as high as major disabilities)	0.75	(0.5, 0.75, 1)
Very High (if the relative risk occurs, the resultant injury level of the workers equals fatalities)	1	(0.75, 1, 1)

**Table 4:** Linguistic variables for Exposure.

Scales (Definitions)	Normalized Value	TFNs
Very low (the respective worker is hardly ever exposed to the relative risk throughout the whole construction phase)	0	(0, 0, 0.25)
Low (the respective worker is seldom exposed to the relative risk throughout the whole construction phase)	0.25	(0, 0.25, 0.5)
Medium (the respective worker is usually exposed to the relative risk throughout the whole construction phase)	0.5	(0.25, 0.5, 0.75)
High (the respective worker is mostly exposed to the relative risk throughout the whole construction phase)	0.75	(0.5, 0.75, 1)
Very High (the respective worker is always exposed to the relative risk throughout the whole construction phase)	1	(0.75, 1, 1)

**Table 5:** Linguistic variables for Detectability.

Scales (Definitions)	Normalized Value	TFNs
Very high (the occurrence of relative risk can certainly be detected by the respective worker)	0	(0, 0, 0.25)
High (the occurrence of relative risk can easily be detected by the respective worker)	0.25	(0, 0.25, 0.5)
Medium (the occurrence of relative risk can moderately be detected by the respective worker)	0.5	(0.25, 0.5, 0.75)
low (the occurrence of relative risk is difficult to be detected by the respective worker)	0.75	(0.5, 0.75, 1)
Very low (the occurrence of relative risk is extremely difficult to be detected by the respective worker)	1	(0.75, 1, 1)

**Table 6:** Output Linguistic Variables.

Risk Magnitude	Value	Normalized Value
Negligible (Ng)	1 ≤ RM < 16	0 ≤ RM < 0.002
Minor	16 ≤ RM < 81	0.002 ≤ RM < 0.012
Major	81 ≤ RM < 256	0.012 ≤ RM < 0.038
Critical	256 ≤ RM ≤ 6561	0.038 ≤ RM < 1

**Table 7:** The crucial safety risks along with their descriptions.

Safety Risks (Code)	Description
Being shocked (SR1)	The workers might be electrocuted that results from being into contact with power lines
Being conflagrated (SR2)	There is a risk of conflagration for the workers working near flammable objects
Being trapped (SR3)	The workers working in close contact with heavy machineries can be entrapped in/between multiple equipment
Fall from height (SR4)	Fall from the suspended or unprotected platform to the lower level
Slip on the floor (SR5)	When the surface on which the respective workers are carrying out task is slippery, they might fall on the floor
Being struck against objects (SR6)	The workers might get struck against any objects, if the materials are not stocked properly on site
Being struck by falling objects (SR7)	The workers working beneath the area where a major construction activity is being undertaken might get severely injured resulting from falling objects
Thermal burn (SR8)	The construction workers might get injured by the contact with hot objects (e.g., hot asphalt and/or tar)
Cut-in (SR9)	Cut-in can be caused when the workers’ body are punctured by the nipping points of machineries
Drowning (SR10)	It stems from falling into river or tank
Spinal disc injury (SR11)	The workers’ spinal disc might get stiffed and/or damaged, resulting from lifting heavy weights
Wrist damages (SR12)	It occurs when the median nerve inside the workers’ wrist is compressed, which results from using vibrate tools for a long period of time
Damages to the tendons (SR13)	It is pertaining to the repetition of the movement of a particular tendon
Stenosing tenosynovitis (SR14)	It is associated with the inflammatory tendons of the workers’ fingers, resulting from gripping the trigger of a power tool for a long period of time
Neck stiffness (SR15)	Damages to the neck of workers that stems from looking up for a prolonged duration
White Finger Disease (SR16)	It is induced by the numbness and tingling of the workers’ fingers, due to working with vibrating hand tools
Mental perturbation (SR17)	Mental disorders occurred to the workers, resulting from the exposure to constant and loud noise for a long period of time
Fatigue (SR18)	It is associated with being involved in heavy construction activities as well as prolonged working hours
Thoracic Outlet Syndrome (SR19)	It is concerned with the reduced blood flow in the shoulder of respective workers being required to carry out overhead works
Heat stroke (SR20)	It results from working in hot and humid conditions
Dengi fever (SR21)	There is a risk of being stung by Dengi mosquito for the workers working on construction sites, resulting from accumulation of stagnant water
Hyperthermia (SR22)	It occurs when the workers’ blood pressure are increased significantly
Chemical rash (SR23)	When the workers are asked to work with chemicals, there is a risk for to develop dermatitis
Chemical burns (SR24)	Chemical burns befall the construction workers, if they are exposed to dangerous chemical substances (e.g., a corrosive substances Lime, lye, etc.)
Prepatellar bursitis (SR25)	It is associated with an inflammation of the bursa inside the knee of the workers, resulting from the pressure imposed on their knees (e.g., roofers installing the panels on roof)
Chemical eye burns (SR26)	Owing to the exposure of workers’ eyes to the solid or liquid chemicals, their eyes may be irritated
Acute inhalation injury (SR27)	The workers’ lungs can severely be damaged, stemming from exposure to the poisonous gas
Arrhythmia (SR28)	Due to the exposure to the chemical substances, the relative workers may experience abnormal heart rhythms
Being choked (SR29)	The respective workers may encounter problems in breathing, resulting from being exposed to the poisonous gas

**Table 8:** Sample data set that is used for ANFIS input and output (29 input datasets out of total 203)

NO. of Data Set	Safety Risks	P	Normalized Value	S	Normalized Value	D	Normalized Value	E	Normalized Value	Normalized Magnitude
1	SR1	5	0.5	9	1	5	0.5	5	0.5	0.171
2	SR2	3	0.25	9	1	5	0.5	3	0.25	0.062
3	SR3	7	0.75	7	0.75	7	0.75	7	0.75	0.366
4	SR4	5	0.5	9	1	1	0	7	0.75	0.048
5	SR5	5	0.5	5	0.5	7	0.75	7	0.75	0.187
6	SR6	3	0.25	3	0.25	9	1	5	0.5	0.062
7	SR7	7	0.75	5	0.5	3	0.25	7	0.75	0.112
8	SR8	3	0.25	3	0.25	3	0.25	5	0.5	0.020
9	SR9	1	0	3	0.25	7	0.75	5	0.5	0.016
10	SR10	1	0	3	0.25	3	0.25	3	0.25	0.004
11	SR11	7	0.75	5	0.5	7	0.75	9	1	0.336
12	SR12	3	0.25	3	0.25	7	0.75	3	0.25	0.029
13	SR13	3	0.25	3	0.25	7	0.75	3	0.25	0.029
14	SR14	3	0.25	3	0.25	7	0.75	3	0.25	0.029
15	SR15	5	0.5	5	0.5	7	0.75	3	0.25	0.080
16	SR16	1	0	1	0	7	0.75	3	0.25	0.003
17	SR17	1	0	3	0.25	9	1	1	0	0.004
18	SR18	5	0.5	1	0	7	0.75	5	0.5	0.027
19	SR19	1	0	1	0	7	0.75	3	0.25	0.003
20	SR20	1	0	3	0.25	7	0.75	3	0.25	0.009
21	SR21	1	0	1	0	7	0.75	1	0	0.001
22	SR22	1	0	1	0	7	0.75	1	0	0.001
23	SR23	5	0.5	3	0.25	5	0.5	5	0.5	0.057
24	SR24	3	0.25	1	0	5	0.5	5	0.5	0.011
25	SR25	1	0	3	0.25	7	0.75	3	0.25	0.009
26	SR26	5	0.5	5	0.5	7	0.75	5	0.5	0.133
27	SR27	3	0.25	3	0.25	7	0.75	3	0.25	0.029
28	SR28	1	0	3	0.25	7	0.75	3	0.25	0.009
29	SR29	1	0	3	0.25	7	0.75	3	0.25	0.009

**Table 9:** ANFIS structure and training parameters.

Number of layers	5
Number of inputs fed to the model	4
Number of input data set	203
Number of output data set	1
Membership functions	Triangular fuzzy numbers
Learning rules	Least squares estimation
Epoch	12

**Table 10:** Risk magnitudes and classifications of safety risks related to testing data sets and aggregation of all data sets.

Safety Risks	Actual Risk Magnitude (Twenty-Nine Test Data Set)	Predicted Risk Magnitude (Twenty-Nine Test Data Set)	Error	Aggregated Risk Magnitude (whole Test Data Set)	Risk Magnitude Classification (Whole Test Data Set)	Actions to Be Taken	Ranks
**SR1**	0.133	0.137	0.004	0.119	Critical	Elimination	3
**SR2**	0.187	0.187	0.000	0.109	Critical	Elimination	6
**SR3**	0.261	0.274	0.013	0.122	Critical	Elimination	2
**SR4**	0.187	0.187	0.000	0.115	Critical	Elimination	4
**SR5**	0.080	0.080	0.000	0.085	Critical	Elimination	7
**SR6**	0.029	0.029	0.000	0.047	Critical	Elimination	12
**SR7**	0.111	0.111	0.000	0.141	Critical	Elimination	1
**SR8**	0.004	0.004	0.000	0.033	Major	Mitigation	14
**SR9**	0.029	0.029	0.000	0.030	Major	Mitigation	16
**SR10**	0.034	0.034	0.000	0.015	Major	Mitigation	24
**SR11**	0.202	0.125	0.077	0.112	Critical	Elimination	5
**SR12**	0.062	0.000	0.062	0.084	Negligible	Acceptance	28
**SR13**	0.037	0.037	0.000	0.053	Major	Mitigation	11
**SR14**	0.080	0.080	0.000	0.031	Major	Mitigation	15
**SR15**	0.048	0.048	0.000	0.039	Critical	Elimination	13
**SR16**	0.016	0.016	0.000	0.007	Minor	Mitigation	27
**SR17**	0.007	0.000	0.007	0.018	Major	Mitigation	23
**SR18**	0.133	0.133	0.000	0.082	Critical	Elimination	8
**SR19**	0.012	0.012	0.000	0.010	Minor	Mitigation	26
**SR20**	0.080	0.080	0.000	0.064	Critical	Elimination	10
**SR21**	0.020	0.020	0.000	0.019	Major	Mitigation	21
**SR22**	0.029	0.029	0.000	0.029	Major	Mitigation	18
**SR23**	0.034	0.034	0.000	0.030	Major	Mitigation	17
**SR24**	0.009	0.009	0.000	0.004	Major	Mitigation	22
**SR25**	0.001	0.001	0.000	0.020	Major	Mitigation	20
**SR26**	0.048	0.048	0.000	0.080	Critical	Elimination	9
**SR27**	0.048	0.000	0.048	0.064	Negligible	Acceptance	28
**SR28**	0.019	0.019	0.000	0.026	Major	Mitigation	19
**SR29**	0.007	0.007	0.000	0.011	Minor	Mitigation	25

**Table 11:** Comparisons between the accuracy of ANFIS and Linear Regression Method (LRM).

Performance Measures	ANFIS	LRM
**RMSE**	0.0843	0.0984
**MAPE**	0.1839	0.2034
**$R^{2}$**	0.9864	0.9137

## Appendix A

In this appendix, a sample of designed ANFIS-based survey is attached. The survey has two sections as follows.

### Appendix A.1. Demographic of Experts

#### Appendix A.1.1. Profile Background

Part A: Kindly indicate only one of the following as your most frequent role:

Contractor	☐
Sub-contractor	☐
Project manager	☐
Safety officer	☐
Construction manager	☐
Site supervisor	☐
Site manager	☐
Quantity surveyor	☐
Operator	☐
Technician	☐

Part B: Kindly indicate how long have you been working in the above-mentioned position?

Between 5 and 10 years	☐
Between 10 and 15 years	☐
Between 15 and 20 years	☐
More than 20 years	☐

#### Appendix A.1.2. Safety Risks Assessment Questionnaire

In this section, please kindly assign the listed linguistic variables to each safety risk identified with respect to the selected four risk parameters.

**Safety Risks (Code)**	**Probability**	**Severity**	**Exposure**	**Detectability**
Being shocked				
Being conflagrated				
Being trapped				
Fall from height				
Slip on the floor				
Being struck against objects				
Being struck by falling objects				
Thermal burn				
Cut-in				
Drowning				
Spinal disc injury				
Wrist damages				
Damages to the tendons				
Stenosing tenosynovitis				
Neck stiffness				
White Finger Disease				
Mental perturbation				
Fatigue				
Thoracic Outlet Syndrome				
Heat stroke				
Dengi fever				
Hyperthermia				
Chemical rash				
Chemical burns				
Prepatellar bursitis				
Chemical eye burns				
Acute inhalation injury				
Arrhythmia				
Being choked				
